# Supplementary material for: Outlier response to anti-PD1 in uveal melanoma reveals germline MBD4 mutations in hypermutated tumors
Source: Nat Commun. 2018 May 14;9:1866. doi: 10.1038/s41467-018-04322-5 (PMC5951831; doi:10.1038/s41467-018-04322-5)
Supplement: Supplementary file 1 — Supplementary Information [file 41467_2018_4322_MOESM1_ESM.pdf]

Supplementary Figures

"Outlier response to anti-PD1 in uveal melanoma reveals germline MBD4 mutations in hypermutated tumors."

Rodrigues et al.

This file contains Supplementary Figures 1-5.

Supplementary Figure 1. Tumor evolution on CT-scan. Axial computed tomography images of UVM_IC metastases before and after 10 months of pembrolizumab infusions illustrating the evolution of the disease.

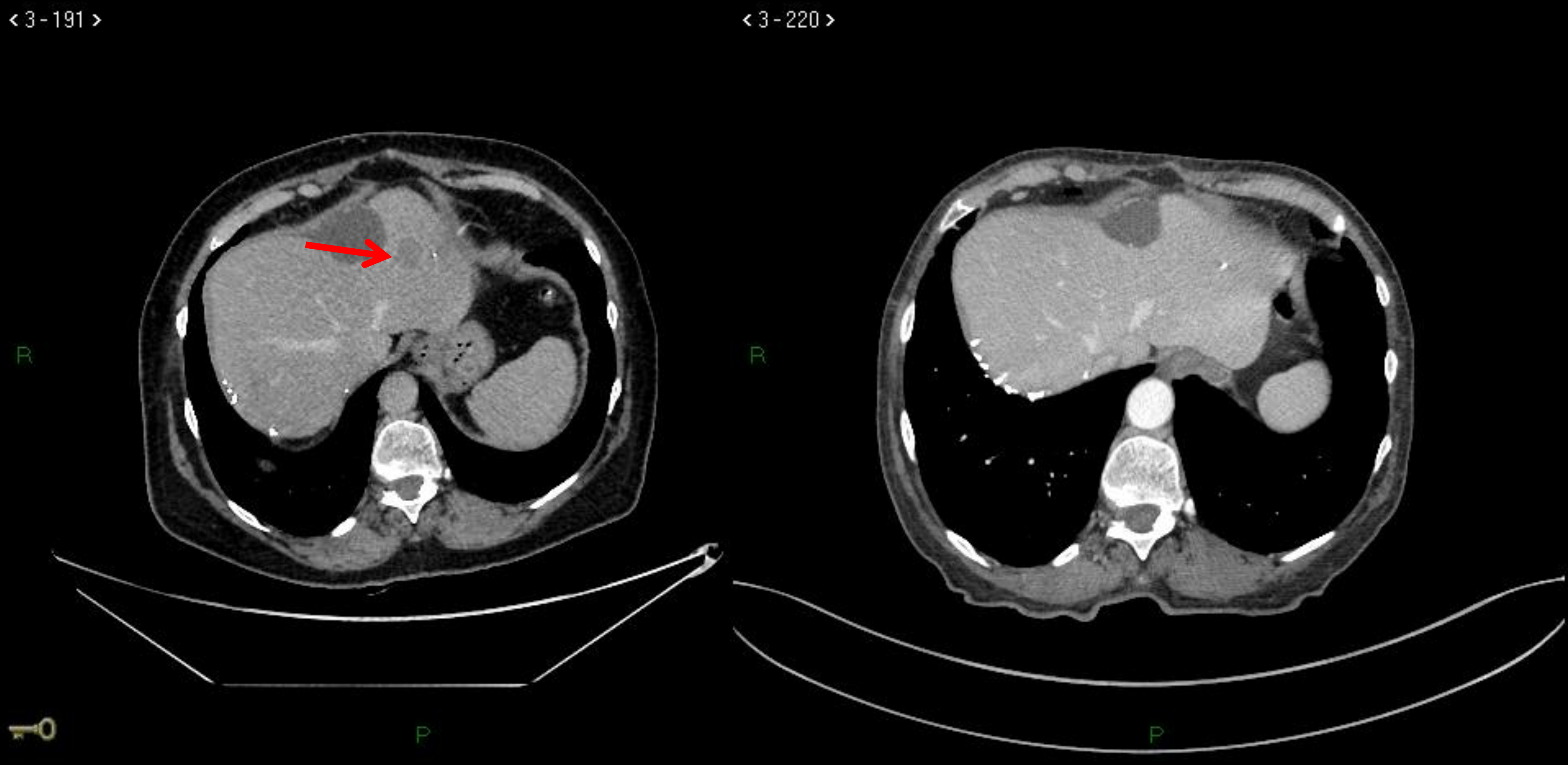

Before initiation of Pembrolizumab

After 10 months of Pembrolizumab

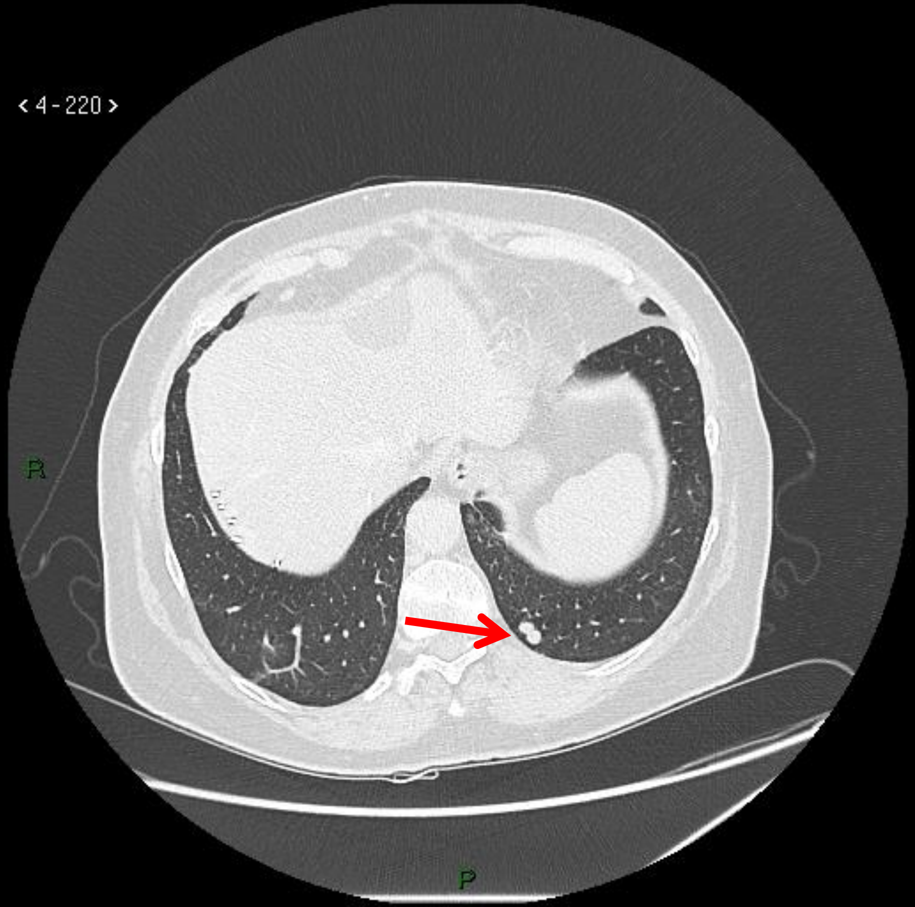

Before initiation of Pembrolizumab

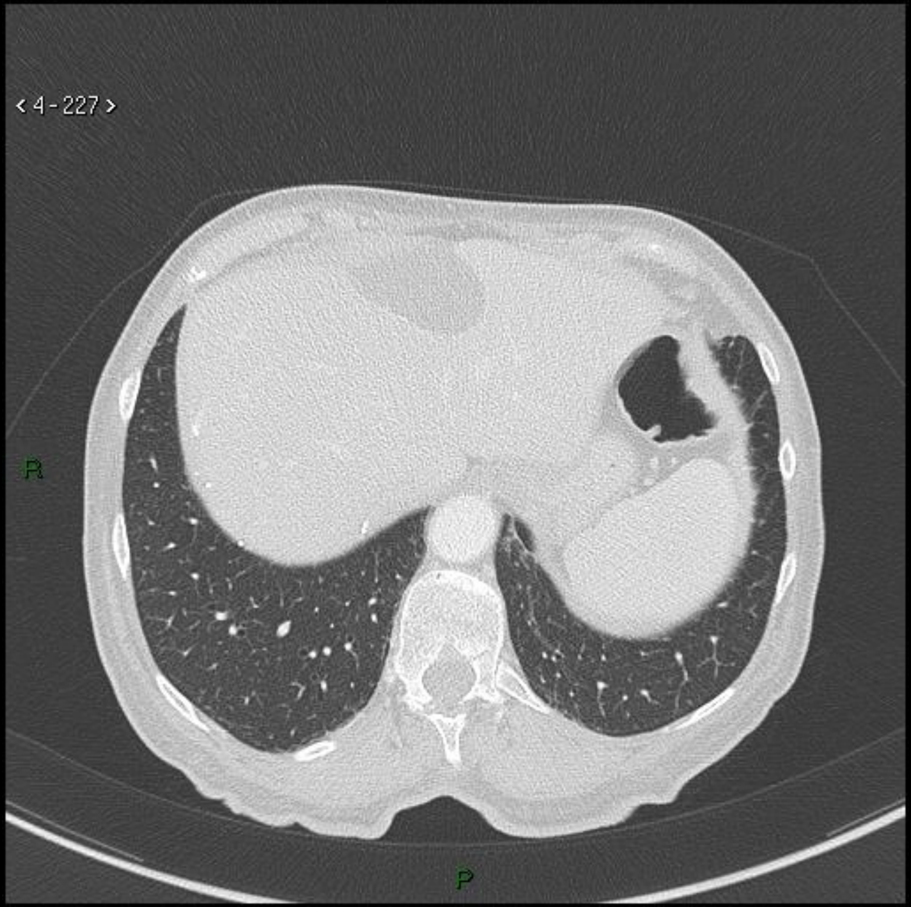

After 10 months of Pembrolizumab

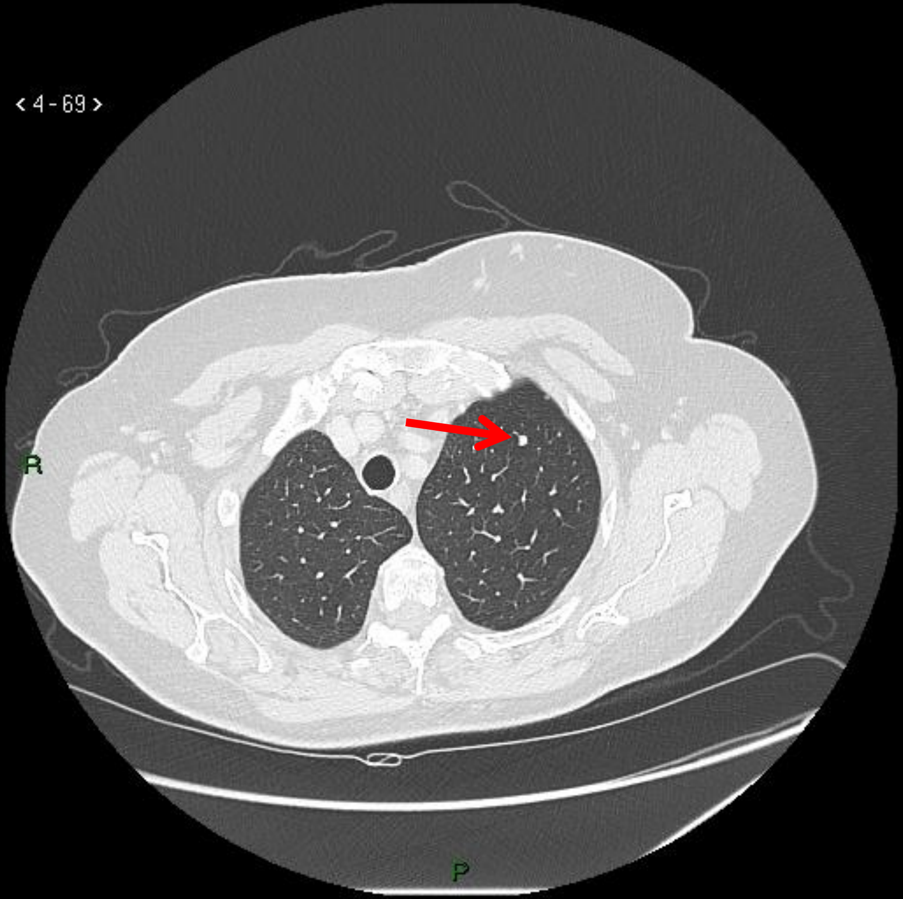

Before initiation of Pembrolizumab

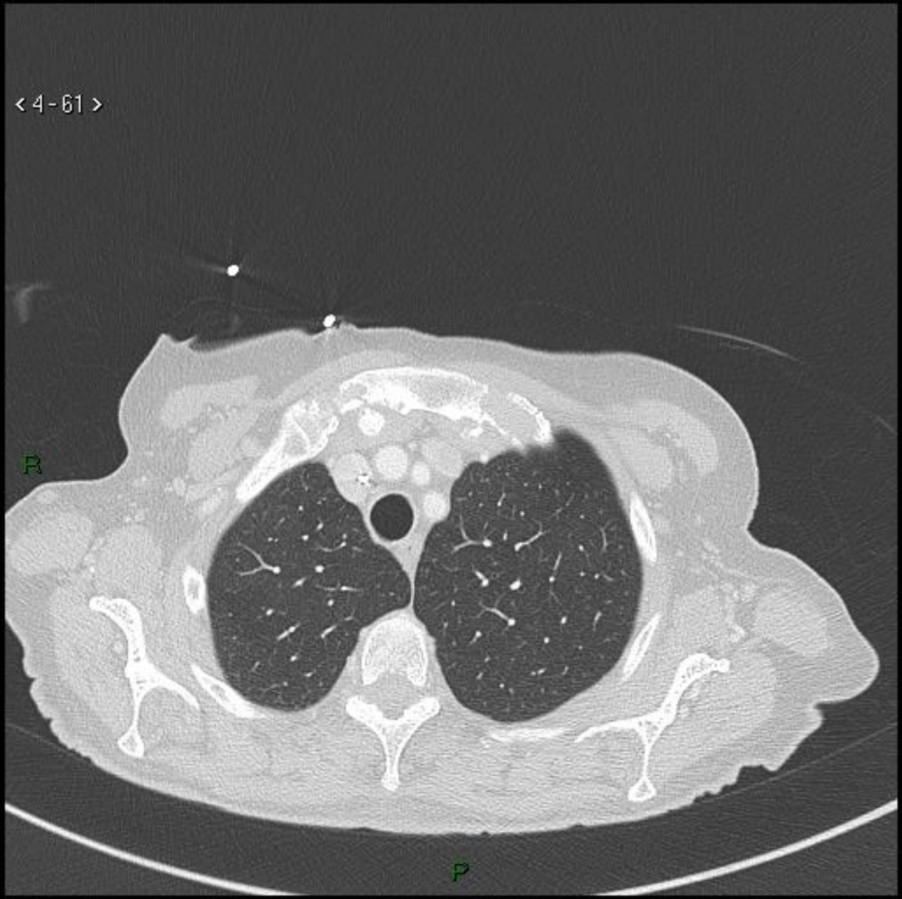

After 10 months of Pembrolizumab

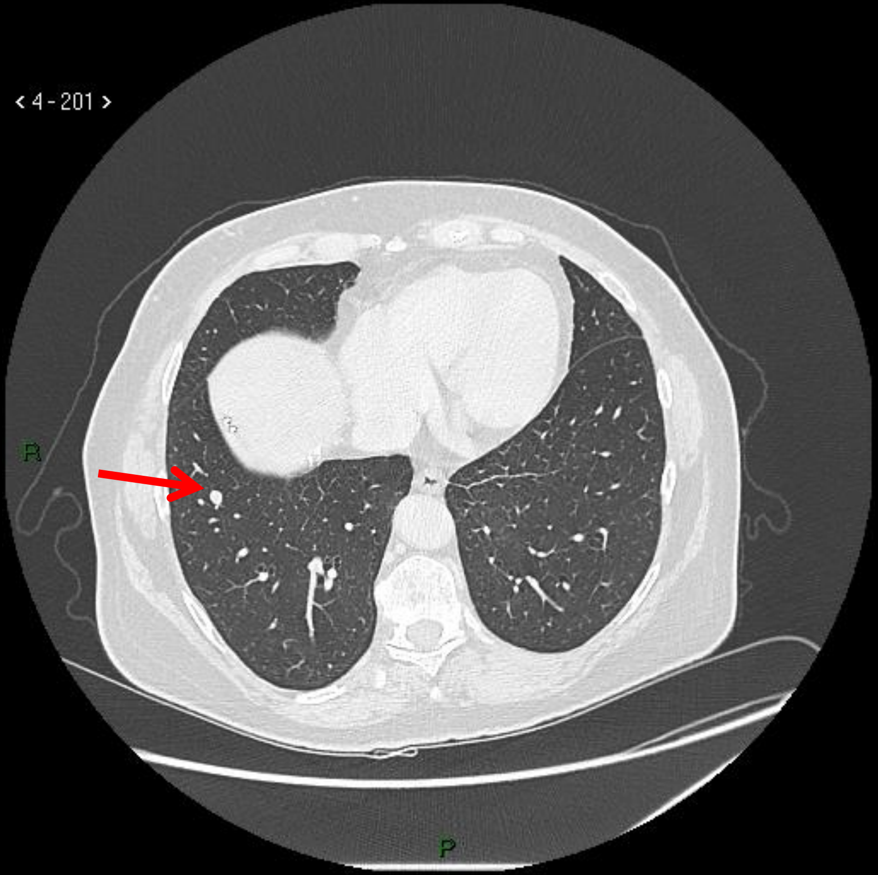

Before initiation of Pembrolizumab

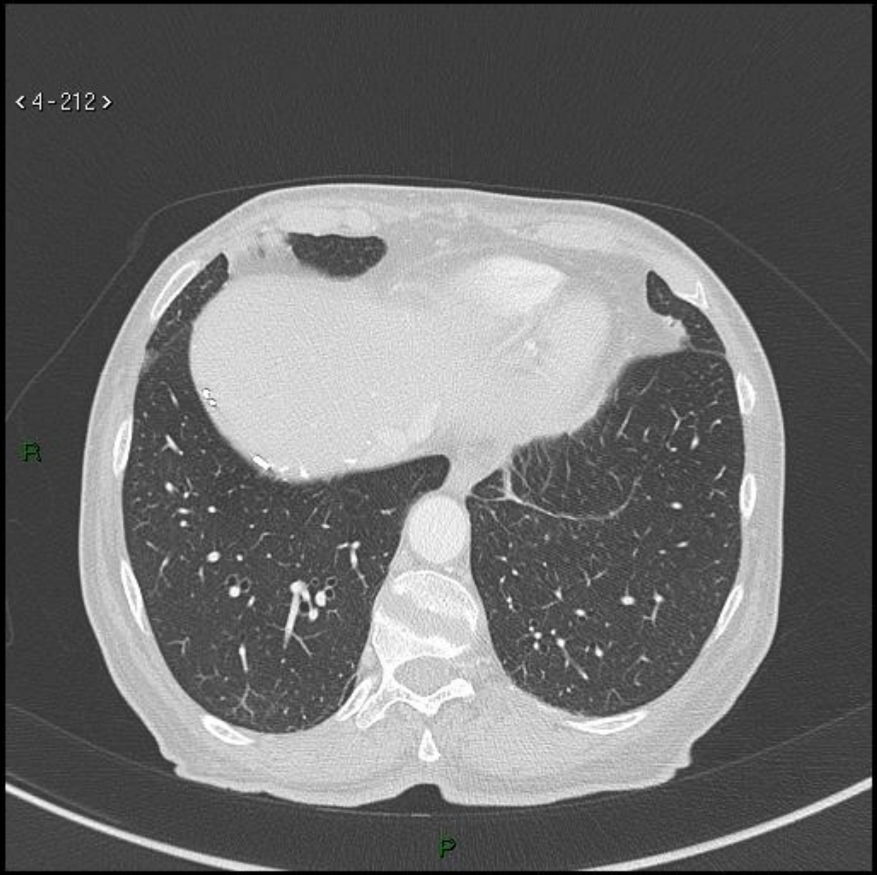

After 10 months of Pembrolizumab

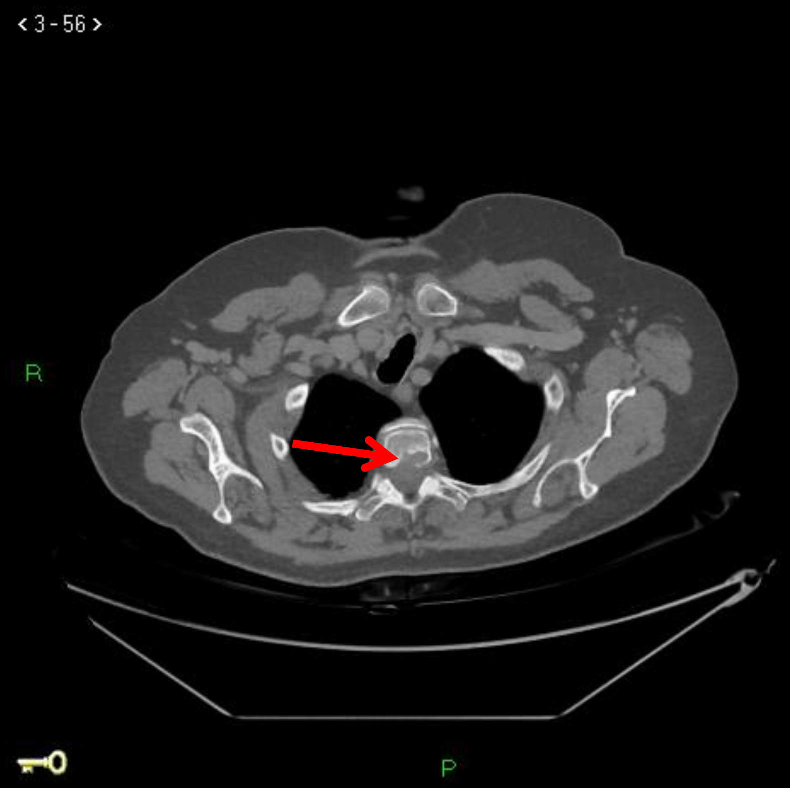

Before initiation of Pembrolizumab

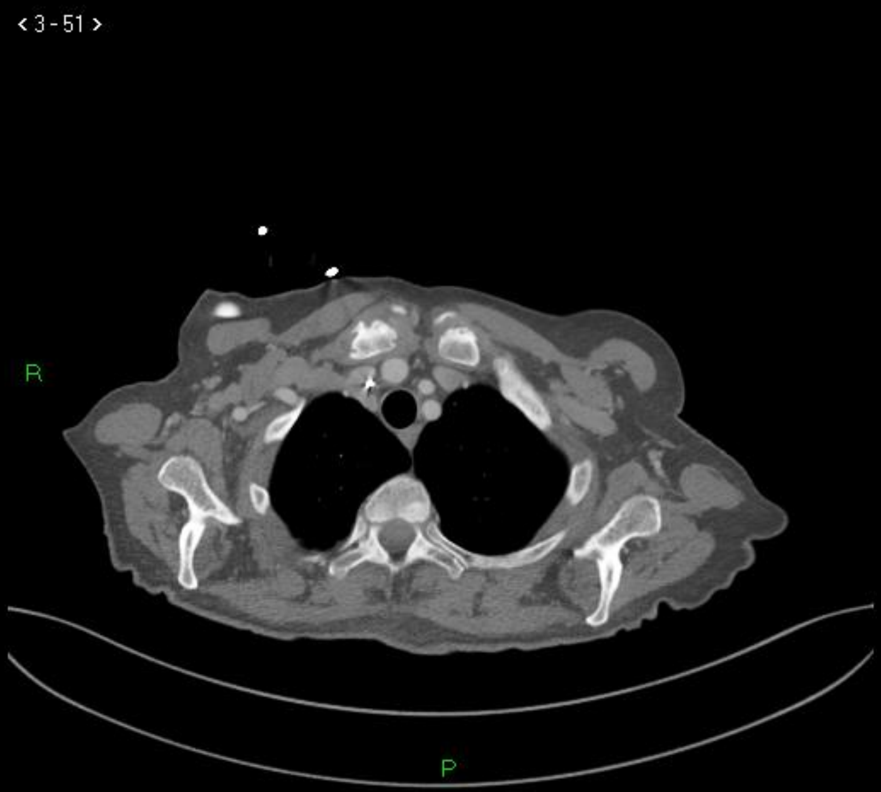

After 10 months of Pembrolizumab

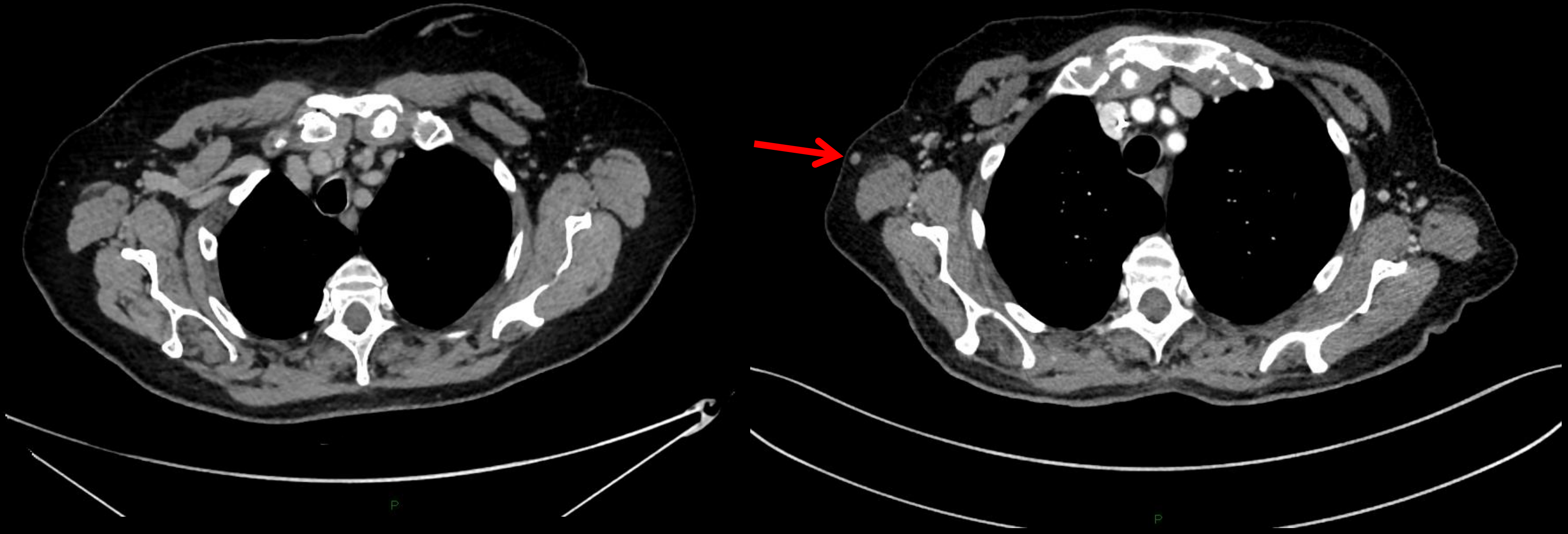

Before initiation of Pembrolizumab

After 10 months of Pembrolizumab

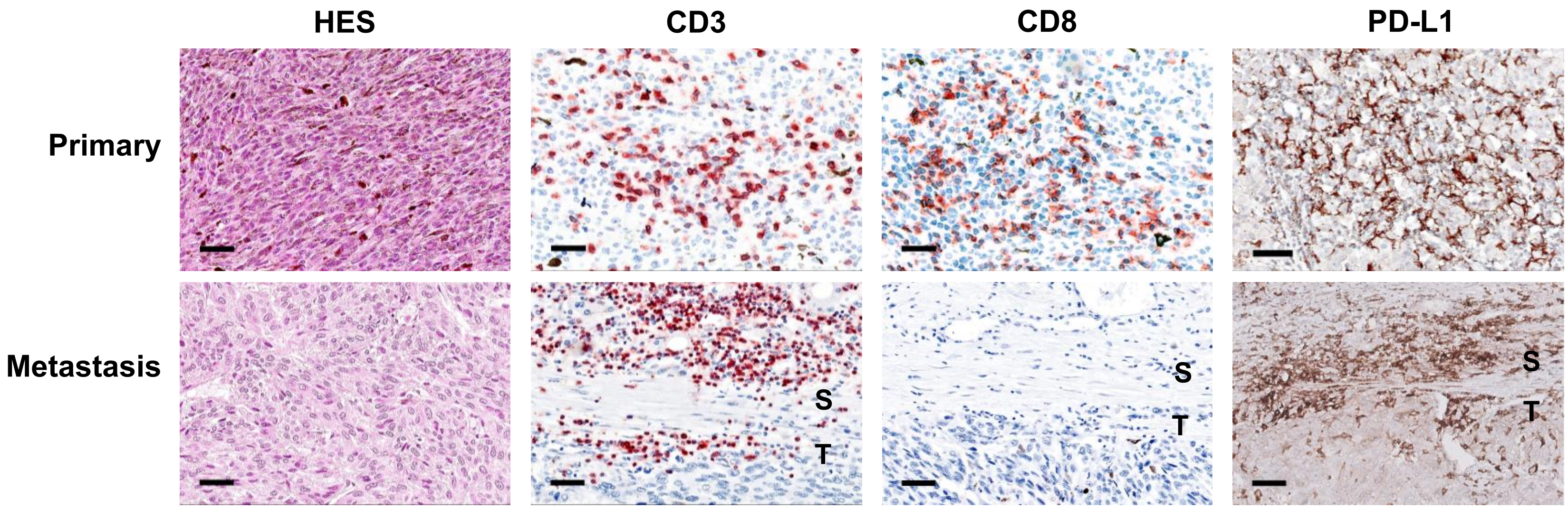

Supplementary Figure 2. Spontaneous immune response against metastatic uveal melanoma in UVM_IC.
Comparison of histopathological and immunohistochemical (IHC) findings in the primary uveal melanoma and liver metastasis prior to pembrolizumab. Hematoxylin eosin staining (HES) pictures illustrate the spindled melanoma cells contingent in primary tumor and an admixture of spindle cells and epithelioid cells in metastasis. A moderate infiltration by Tumor-Infiltrating Lymphocytes (TILs) in the primary tumor is evidenced by anti-CD3 IHC. A moderate tumor infiltrate and a high peri-metastatic T-cell infiltrate is seen at the edge of the metastasis (S: stroma; T: tumor). CD8 IHC shows a moderate infiltration by CD8+ TILs in the primary tumor and no intra- or peri-metastatic CD8+ TILs. PD-L1 membranous expression is present in ~30% of primary tumor cells and ~20% of metastatic tumor cells, while 90% of peri-metastatic immune cells expressed PD-L1. All the scale bars represent 50 µm.

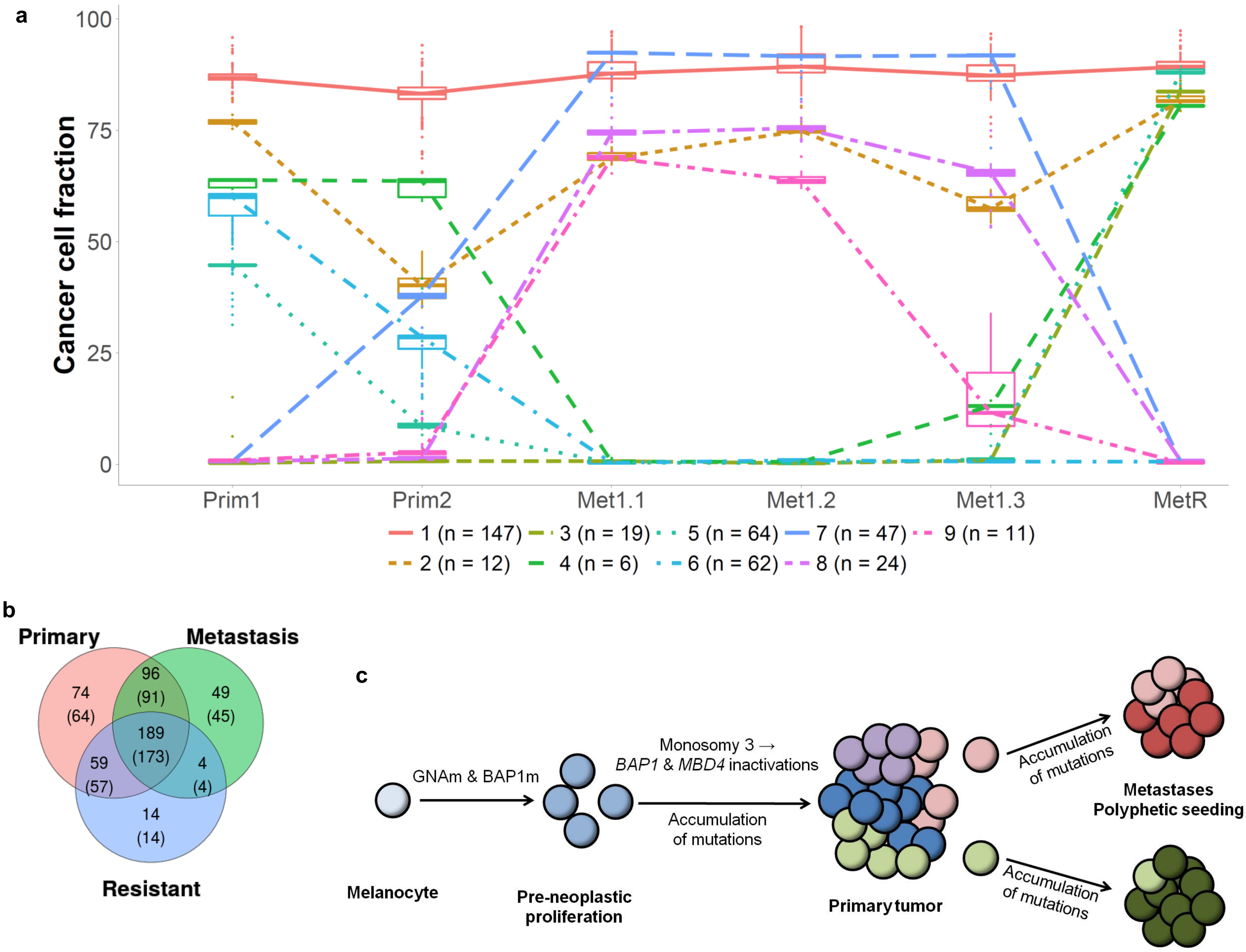

Supplementary Figure 3. *MBD4*-related mutator process is a continuous process. (a) Tumor clonal evolution was inferred with the Pyclone software from grouped sets of somatic single nucleotide variants SNVs found on each samples. Each line correspond to a grouped set of SNVs with similar evolution. Number of SNVs in each group is indicated below. The lower and upper hinges correspond to the first and third quartiles (the 25th and 75th percentiles). The upper whisker extends from the hinge to the largest value no further than 1.5 * IQR from the hinge (where IQR is the inter-quartile range, or distance between the first and third quartiles). The lower whisker extends from the hinge to the smallest value at most 1.5 * IQR of the hinge. Data beyond the end of the whiskers are called "outlying" points and are plotted individually. (b) Venn diagram presenting the number of total SNVs (above) *versus* CpG>TpG only (below, between parentheses). (c) Model of progression in *MBD4*-related uveal melanoma.

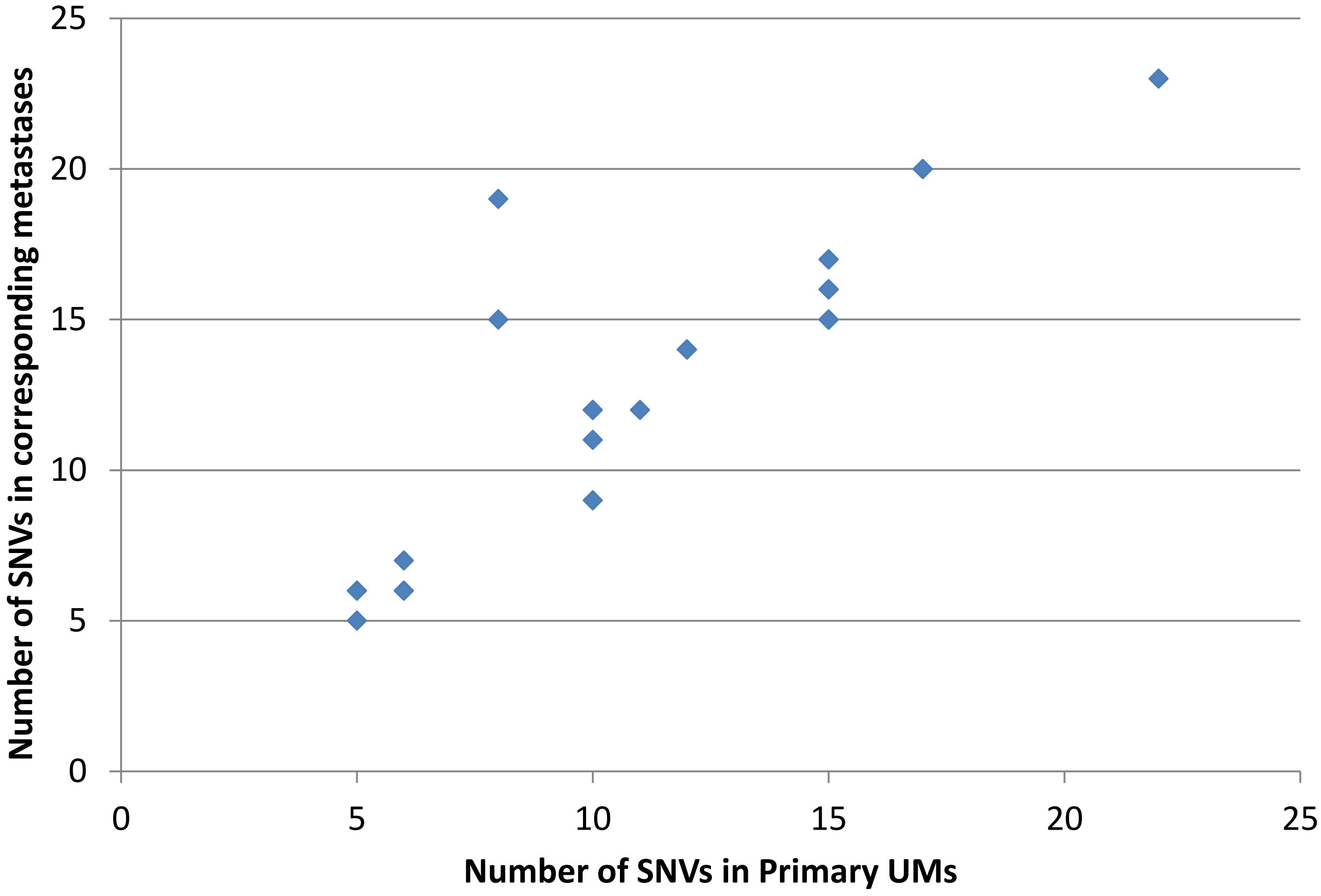

Supplementary Figure 4. Number of SNVs in primary uveal melanomas (UMs) *versus* corresponding metastases. We sequenced 11 trios (germline, primary UM and metastases from same patient). The number of SNVs found in primary tumor is plotted on the X-axis, while the number of SNVs found in corresponding metastatic samples is plotted on the Y-axis.

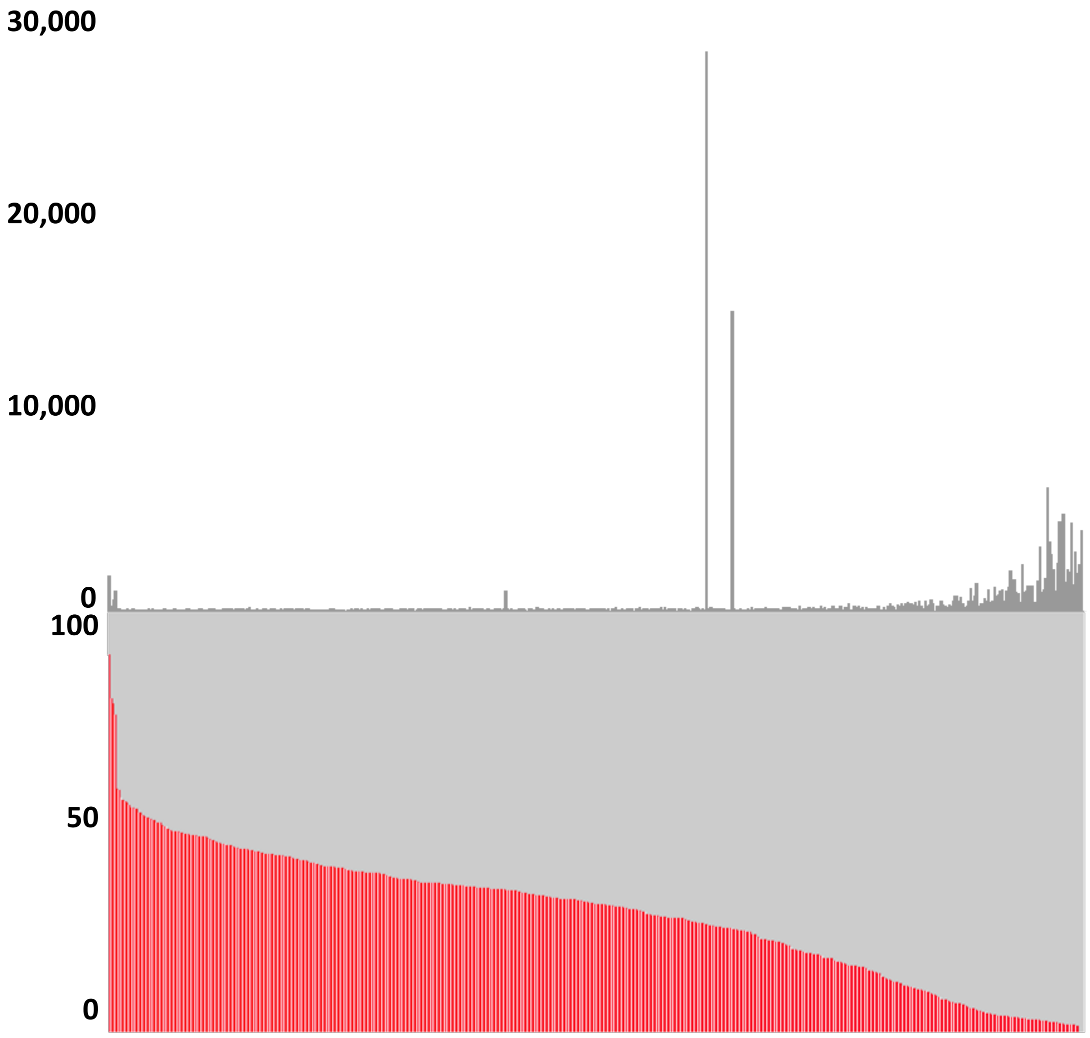

Supplementary Figure 5. Number and spectrum of mutations in the whole glioblastoma TCGA series. Above total number of mutations in 496 tumors. Below, proportion of CpG>TpG mutations in red *versus* all other mutations in grey.
