# Supplementary material for: Outlier response to anti-PD1 in uveal melanoma reveals germline MBD4 mutations in hypermutated tumors
Source: Nat Commun. 2018 May 14;9:1866. doi: 10.1038/s41467-018-04322-5 (PMC5951831; doi:10.1038/s41467-018-04322-5)
Supplement: Supplementary file 3 — Description of Additional Supplementary Files [file 41467_2018_4322_MOESM3_ESM.pdf]

Description of Additional Supplementary Files

Supplementary Data 1: List and annotations of tumors with more than 200 single nucleotide variants (SNVs) extracted from the whole TCGA series.
